# BJPsych Bulletin In Memoriam List 2025

**DOI:** 10.1192/bjb.2024.119

**Published:** 2025-02

- Abrahamson, David, Fellow ~ Over 40 Years member
- Al-Nufoury, Maysan, Member
- Ayuba, Larry Nanjul, Member
- Berlyne, Neville, Fellow ~ Over 40 Years member
- Black, Dorothy Florence Mary, Fellow ~ Over 40 Years member
- Brooks, Simon Alan Laurence, Fellow ~ Over 40 Years member
- Browne, Ivor William, Fellow ~ Over 40 Years member
- Bulmer, David Rennie, Member
- Burman, Anthony Stuart, Member ~ Over 40 Years member
- Byrne, Alan Paul, Member
- Causton, John Allan, Member ~ Over 40 Years member
- Choudry, Ansar Ali, Member
- Cooper, John Edward, Fellow ~ Over 40 Years member
- Cooray, Sherva Elizabeth, Fellow ~ Over 40 Years member
- Crossley, Robert Brian, Fellow ~ Over 40 Years member
- Davies, Gaius, Fellow ~ Over 40 Years member
- Davies, Joy, Member ~ Over 40 Years member
- Devine, Roy, Fellow ~ Over 40 Years member
- Farhoumand, Noushin, Fellow ~ Over 40 Years member
- Gander, Derek Reginald, Fellow ~ Over 40 Years member
- Godber, Colin, Fellow ~ Over 40 Years member
- Goldberg, David Paul Brandes, Honorary Fellow Previous Member
- Hale, Anthony Stephen, Member
- Harper, Max Alan, Fellow
- Hartley, Eleanor Dorothy Margaret, Member ~ Over 40 Years member
- Harwin, Brian Gordon, Fellow ~ Over 40 Years member
- Humphreys, Martin Sydney, Fellow
- Ijete, Onikepe, Affiliate
- Jolley, David James, Fellow ~ Over 40 Years member
- Knights, Angela Clayton, Fellow ~ Over 40 Years member
- Kosorinova, Eva, Affiliate
- Marshall, Philip David, Member
- Merskey, Harold, Fellow ~ Over 40 Years member
- Millar, Vivien Ann, Member ~ Over 40 Years member
- Minton, Derek Bernard, Member ~ Over 40 Years member
- Norris, William Anderson, Fellow ~ Over 40 Years member
- Ogbomor, Frank Ifeanyi, Affiliate
- O'Kane, Aideen Mary, Member
- Pauleau, Nitza, Affiliate
- Pavlovic, Ruth Yvonne, Member
- Perez-Montejano, Raul, Specialist Associate
- Piper, Mary Evelyn, Member ~ Over 40 Years member
- Ramakrishnan, Vellepur Hanumanthappa, Member
- Robertson, Alan Roxburgh, Member
- Rogers, Paul, Mental Health Associate
- Sarkar, Paresh Nath, Member ~ Over 40 Years member
- Schelhase, Monique Therese, Affiliate
- Smith, Michael Kenny, Member ~ Over 40 Years member
- Tang, Wai-Nang Orlando, Fellow
- Tough, Harry Gordon, Fellow ~ Over 40 Years member
- Warshow, Mbarak Majid, Member
- Wooding, Clifford Desmond, Member

